# Big lessons from the little *Akkermansia muciniphila* in hepatocellular carcinoma

**DOI:** 10.3389/fimmu.2025.1524563

**Published:** 2025-02-14

**Authors:** Yanguang Yang, Xinli Shi

**Affiliations:** ^1^ Laboratory of Integrated Medicine Tumor Immunology, Shanxi University of Chinese Medicine, Taiyuan, China; ^2^ Department of Pathobiology and Immunology, Hebei University of Chinese Medicine, Shijiazhuang, China

**Keywords:** hepatocellular carcinoma, *Akkermansia muciniphila*, immunotherapy, traditional Chinese medicine, intestinal flora

## Abstract

Hepatocellular carcinoma (HCC) is the most frequently occurring type of liver tumor and is considered one of the most common primary malignant neoplasms. The prognosis for HCC is dismal because of its complicated etiology and high level of medication resistance. Immunotherapy is presently regarded as one of the most effective therapeutic options for HCC; nevertheless, because of the disturbance of intestinal flora, immunotherapy shows low antitumor efficacy. An increasing body of research indicates that intestinal flora, particularly *Akkermansia muciniphila* (*A. muciniphila*), is vital for the treatment of tumors. Studies have demonstrated that the diminished effectiveness of immunotherapy in cancer patients is associated with a reduction in *A. muciniphila* levels, suggesting that increasing *A. muciniphila* levels significantly enhance the efficacy of immunotherapy. *A. muciniphila* functions as a gut probiotic and can treat and prevent a wide range of illnesses, including cancer. Consequently, preserving *A. muciniphila* abundance is enough to prevent and lower the danger of developing cancer disorders. In this review, we critically evaluate the current body of research on *A. muciniphila*, with a primary focus on its biological properties and functions. The different illnesses that *A. muciniphila* treats were then discussed, particularly the way it works with liver cancer. This review aims to give a novel treatment plan for patients with HCC as well as a theoretical foundation for improving HCC immunotherapy.

## 1 Introduction

Liver cancer is the most prevalent type of primary tumor, ranking third in cancer mortality and sixth in incidence, with hepatocellular carcinoma (HCC) being its main type (1). Presently, hepatectomy is the main radical treatment for patients with early liver cancer, and radiofrequency ablation can be selected for patients who cannot be operated (2). Transcatheter arterial chemoembolization (TACE) is mainly used in patients with mid-term HCC (2). When TACE treatment fails in monkeys, systemic treatment will be recommended (3). Systemic treatment is suitable for most patients with HCC in intermediate and advanced stages. Current first-line treatment includes atezolizumab combined with bevacizumab, sorafenib, and lenvatinib (2). In recent years, the advent of immunotherapies, including immune checkpoint inhibitors (ICIs), has brought new hope to cancer patients, such as anti-programmed cell death 1 (PD-1)/L1 antibodies, anticytotoxic T lymphocyte-associated antigen-4 (CTLA-4) antibodies, and anti-lymphocyte-activating gene-3 (LAG3) antibodies (4–6). A study reported that the median overall survival (mOS) of atezolizumab combined with bevacizumab was longer than that of sorafenib (7). Although much work has been done to enhance responses and eliminate toxicities, the response of HCC patients to ICI remains unsatisfactory.

In recent years, there is growing evidence that there is a correlation between intestinal flora and the efficacy of tumor immunotherapy (5, 8). Studies have shown that after receiving ICI treatment, analysis of fecal samples from patients has found a decrease in the diversity and richness of intestinal flora, which is associated with low response rates and survival rates in patients (9, 10). For example, disorders of intestinal flora may promote tumor progression, through mechanisms including the production of toxic metabolites, induction of an inflammatory environment, and suppression of antitumor immunity (10). Thus, in mouse models of tumors, mice receiving ICI had reduced antitumor effects after antibiotic treatment (11). However, intestinal flora regulates the efficacy and toxicity of cancer immunotherapy, and the most researched is improving immunotherapy and its associated side effects (10). The most renowned study involved the transplantation of fecal matter from patients who had shown a positive response to immunotherapy into germ-free mice, revealing that these mice exhibited enhanced immunity, significantly inhibited tumor growth, and enhanced the effectiveness of anti-PD-L1 therapy (12, 13). Therefore, targeted regulation of intestinal flora is one of the effective ways to improve the efficacy of immunotherapy in tumors.

*Akkermansia muciniphila* (*A. muciniphila*), a bacterium that inhabits the intestinal mucus layer, is recognized as the most prevalent lytic bacteria in the intestines of healthy hosts and plays a crucial role in maintaining the intestinal barrier, promoting mucus production, and regulating mucus layer thickness (14, 15). The specific mechanism is that *A. muciniphila* reduces intestinal permeability, increases the expression of tight junction proteins, and increases the number of goblet cells that produce mucins (16–18). Furthermore, *A. muciniphila* has been implicated in the progression of various diseases, including inflammatory bowel diseases (19, 20), aging-related conditions (21), obesity (22, 23), type 2 diabetes (T2D) (24, 25), cardiovascular disorders (26, 27), non-alcoholic fatty liver disease (NAFLD) (28, 29), and cancers (11). Another study showed that whether *A. muciniphila* is live or pasteurized, oral administration is safe for human volunteers (30). In 2021, the European Food Safety Authority (EFSA) affirmed the safety of pasteurized *A. muciniphila* in adults and authorized its usage as a food supplement (31). Consequently, elucidating the molecular and immune response mechanisms of *A. muciniphila* in disease contexts, as well as determining safe supplementation strategies for *A. muciniphila* during disease states, is crucial for the exploration of innovative therapeutic approaches aimed at enhancing prevention and treatment outcomes.

This review seeks to clarify the connection between *A. muciniphila* and HCC, particularly in its role as an adjuvant in immunotherapy, while also elucidating the possible mechanisms behind the antitumor effects of *A. muciniphila*. Therefore, this review seeks to propose an innovative treatment strategy for patients with HCC while providing a theoretical framework to enhance immunotherapy approaches for HCC.

## 2 The background introduction of *A. muciniphila*

*A. muciniphila*, a common intestinal anaerobic bacterium, was first successfully isolated in 2004 (15). It is widely distributed in the intestines of humans and animals and has the largest number in the cecum, accounting for 3%–5% of the human microbiome (15, 32). *A. muciniphila* relies solely on mucin as its primary source of carbon, nitrogen, and energy, thereby supplying energy to epithelial cells (33). The main function of *A. muciniphila* involves the breakdown of mucus, facilitated by a range of mucolytic enzymes encoded in its genetic material (34, 35). In addition, *A. muciniphila* and its functional active components, including metabolites, metabolic enzymes, and outer membrane proteins, effectively improve intestinal barrier function by regulating metabolism and enhancing tight junctions (36–38). Interestingly, *A. muciniphila* commenced colonization and exhibited rapid growth and enrichment in the human intestines during infancy (39). However, its abundance progressively declined with aging and the progression of disease (39, 40). Therefore, *A. muciniphila* supplementation has demonstrated therapeutic benefits for a range of diseases, including liver diseases, colitis, obesity, and diabetes (40). At present, there are many traditional Chinese medicine formulas, individual Chinese medicinal materials, and extracts to increase the number of *A. muciniphila*, including Si-Ni-San (41), Liangxue guyuan yishen decoction (42), wolfberry (43), berberine (44), and *Dendrobium officinale* polysaccharide (45). In summary, increasing the abundance of *A. muciniphila* has become one of the effective treatment measures for many diseases.

### 2.1 Biological characteristics of *A. muciniphila*

*Akkermansia muciniphila* is classified within the *Akkermansia* genus of the *Verrucomicrobia* (15, 34). Moreover, in addition to *A. muciniphila*, this study also includes other species within the genus *Akkermansia*, such as *massiliensis* (46), *biwaensis* (47), *ignis* (48), *durhamii*, and others with less precise descriptions (49). This bacterium is characterized as gram-negative, non-motile, and non-spore-forming, and the long axis of the cell changes with the medium. In the mucin medium, the strain exhibits a diameter of 640 nm and a length of 690 nm; however, in brain–heart infusion (BHI), the diameter increases to 830 nm with a corresponding length of 1 μm (15). It predominantly exists as individual cells or in pairs, with chain formation being a rare occurrence (15). Interestingly, the strictly anaerobic *A. muciniphila* demonstrates a notable capacity for survival when subjected to atmospheric conditions. After 24 h in a standard air environment, its survival rate is observed to be 25%, while after 48 h, this rate declines significantly to merely 1% (33). Studies have demonstrated that *A. muciniphila* can be cultured in various media, including BHI, porcine mucin, human mucin combined with BHI medium, Colombia medium, and tryptic soy broth (15, 50–52). The medium utilized by our research group comprised 2.45 g of BHI, 0.3 g of mucin, 100 ml of a 0.3% L-cysteine solution, and autoclaved distilled water, which were mixed uniformly (43). Furthermore, *A. muciniphila* is cultivated at temperatures ranging from 20°C to 40°C and a pH of 5.5 to 8.0, with optimal growth conditions identified as 37°C and a pH of 6.5 (15). Notably, the bacterial growth is inhibited when the pH falls below 5.5 or exceeds 8.0. In the agar base medium, the *A. muciniphila* colony displayed a circular morphology, appeared white in color, and had a diameter of 0.7 mm (15).

*A. muciniphila* is recognized as one of the most prevalent bacterial species, comprising over 1% of the overall fecal microbiota (53). Fluctuations in its abundance are closely associated with various factors including age, diet, body weight, disease states, and immune status (54). Similar to other intestinal bacteria, *A. muciniphila* predominantly colonizes the outer layer of intestinal mucus; its development is maintained by carbon and nitrogen produced by goblet cells to maintain the dynamic balance of the mucus layer (55–57). A study published in 2007 revealed that *A. muciniphila* is capable of colonizing the intestines of infants early in life, achieving levels comparable to those found in adults within 1 year, and subsequently experiencing a gradual decline in abundance among the elderly population (39). Interestingly, recent studies have revealed that *A. muciniphila* is not only present in the human intestine but is also detected in the nasopharynx and breast milk, where it utilizes human milk oligosaccharides as a carbon source (58–60). This finding offers robust evidence supporting the early colonization of *A. muciniphila* within the intestinal microbiota. Furthermore, *A. muciniphila* is not only colonized in the human intestines but is also present in the intestinal microbiota of various other animals, including rats (61), mice (62), horses (63), rex rabbits (64), guinea pigs (65), calves (66), rock ptarmigans (67), zebrafish (68), and Burmese pythons (69). These findings demonstrate the extensive colonization of *A. muciniphila* across various animal models and provide foundational research to support its applications.

The initial gene sequencing of *A. muciniphila* ATCC BAA-835 was accomplished in 2011, revealing a complete genome comprising a circular chromosome of 2,664,102 bp and encompassing a total of 2,176 predicted protein-coding sequences, which collectively exhibit an overall coding capacity of 88.8% (35). Twenty-two *A. muciniphila* species were isolated from the feces of people in southern China, which had more than 99% consistency with strain ATCC BAA-835, and 12 subtypes were distinguished by enterobacterial repetitive intergenic consensus (ERIC-PCR) (70). Additionally, *A. muciniphila* in the human gut utilized over 2,000 genomes from humans and various other animals for a comprehensive population genomic analysis, resulting in the delineation of five distinct candidate species (71). In 2017, a total of 39 A*. muciniphila* samples derived from human and mice feces were sequenced to elucidate the genomic structure, leading to the identification of three distinct species-level *A. muciniphila* populations (AmI, AmII, and AmIII) through phylogenetic analysis (72). Furthermore, through an integrative approach combining genomic, molecular biology, and traditional microbiological methods, *A. muciniphila* has identified at least four species-level groups within its systems (AmI to AmIV) exhibiting different functional characteristics (73). Subsequent investigations indicated that the AmII strain possessed a gene responsible for vitamin B_12_ synthesis. In the absence of vitamin B_12_, the AmII strain was able to produce acetate and propionate, whereas the AmI strain produced acetate and succinate. Interestingly, the abundance of AmI strains was greatest among children and adolescents; however, antibiotic-treated mice exhibited a higher quantity of AmII and AmIV strains compared to AmI strains (74).

### 2.2 Active compounds and metabolites obtained from *A. muciniphila*

**Figure 1:**
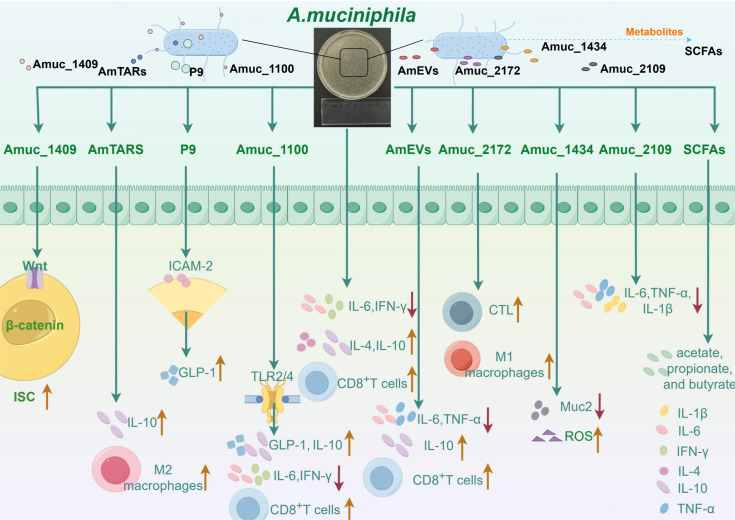
The ways in which *Akkermansia muciniphila* and its active proteins interact with the immune system are complex and varied. *Akkermansia muciniphila* has been shown to reduce the levels of interleukin-6 (IL-6) and interferon-γ (IFN-γ) in mice with abdominal aortic aneurysms. Moreover, *A. muciniphila* simultaneously increased the concentrations of interleukin-4 (IL-4) and interleukin-10 (IL-10), thereby enhancing the capacity of A549 and NCI-H1395 cells to recruit CD8^+^ T cells. Amuc_1100 mitigates inflammatory responses by decreasing IFN-γ and IL-6 levels in both the spleen and pancreas. Furthermore, Amuc_1100 interacts with Toll-like receptor 2 (TLR2) and modestly promotes glucagon-like peptide-1 (GLP-1) production by L cells, thus reinforcing gut barrier function. It also plays a role in the host intestinal immune response through activation of TLR2 and TLR4, leading to increased IL-10 production. Additionally, Amuc_1100 exhibits potent antitumor effects by augmenting both the quantity and functionality of CD8^+^ T cells. Studies suggest that P9 interacts with intercellular adhesion molecule 2 (ICAM-2), leading to the activation of the glucagon-like peptide-1 receptor (GLP-1R) pathway, which in turn enhances GLP-1 production. Moreover, AmTARS facilitates M2 macrophage polarization while specifically interacting with TLR2 to induce IL-10 production. Moreover, Amuc_1409 regulates intestinal stem cell (ISC) activity via Wnt/β-catenin signal transduction pathways. Extracellular vesicles derived from *A. muciniphila* (AmEVs) significantly lower serum TNF-α and IL-6 levels while elevating IL-10 concentrations, thereby ameliorating liver damage and fibrosis in murine models with hepatic injury. Studies have demonstrated that these extracellular vesicles can serve as an immunotherapeutic agent against prostate cancer progression primarily through activating effector states in CD8^+^ T cells alongside promoting macrophage polarization toward an M1 phenotype. Furthermore, Amuc_2172 enhances transcriptional expression as well as secretion levels of heat shock protein 70 (HSP70), subsequently increasing CD8 cytotoxic T lymphocyte (CTL) numbers and activities within colorectal cancer contexts. In another investigation, it was revealed that Amuc_2172 induces M1 macrophage polarization; however, this effect is reversed by puerarin treatment which improves ulcerative colitis outcomes. Lastly, Amuc_1434 promotes apoptosis along with degradation processes involving mucin 2 (Muc2) within LS174T cells while inducing intracellular reactive oxygen species (ROS). Recent findings suggest that Amuc_2109 reduces TNF-α, IL-1β, and IL-6 levels alongside downregulating NLR family pyrin domain 3 (NLRP3) expression while enhancing gut barrier integrity—thereby improving DSS-induced colitis symptoms. Short-chain fatty acids (SCFAs)—the primary metabolites produced by *A. muciniphila*—are predominantly composed of acetate, propionate, and butyrate.

In the contemporary research landscape, an escalating volume of investigations has been centered on *A. muciniphila* and its specific derivatives, encompassing pasteurized *A. muciniphila*, the Amuc series, extracellular vesicles (AmEVs), short-chain fatty acids (SCFAs), and metabolic enzymes (**Figure 1**).

#### 2.2.1 The living and pasteurized *A. muciniphila*

Current research indicates that live *A. muciniphila* modulates the intestinal immune response and fortifies intestinal barrier function, participating in the immune system and enhancing the intestinal barrier through the production of SCFAs and acetate (16, 75). Furthermore, due to its capacity to degrade intestinal mucus, *A. muciniphila* may exert an indirect influence on both innate and adaptive immune responses (76). *A. muciniphila* is frequently correlated with inflammation-related cytokines. Studies reported that during liver injury treatment, *A. muciniphila* combined with inosine increased the number of regulatory T (Treg) but reduced T helper (Th) 1 (77). During lactation, *A. muciniphila* levels in breast milk are positively correlated with TNF-α and IFN-γ levels but negatively correlated with interleukin-10 (IL-10) and interleukin-4 (IL-4) levels (59). In contrast, *A. muciniphila* was observed to decrease the expression of interleukin-33 (IL-33) and the levels of interleukin-6 (IL-6) and IFN-γ in mice with abdominal aortic aneurysm, while simultaneously increasing the concentrations of IL-4 and IL-10 (26). Additionally, our previous research demonstrated that during dihydroartemisinin (DHA) treatment, *A. muciniphila* increased the population of CD8^+^ T cells in both the spleen and tumor niche of liver cancer model mice (78). Similarly, *A. muciniphila* enhances the capacity of A549 and NCI-H1395 cells to recruit CD8^+^ T cells and augments the cytotoxicity of CD8^+^ T cells (79). Interestingly, in Lewis lung cancer mouse models treated with anti-PD-1 therapy, *A. muciniphila* activates the MHC-II–pDC pathway, reduces the expression of CXCL3 in cancer-associated fibroblasts, stimulates CD8^+^ T-cell activation, and enhances their functionality compared to pasteurized *A. muciniphila* and Amuc_1100 (80). The combination of *A. muciniphila* and cisplatin inhibits the growth of lung cancer in mice, with the underlying mechanism involving an increase in serum expression of IFN-γ, IL-6, and TNF-α and a reduction in the number of Treg cells (81). In another study, *A. muciniphila* improved the inflammation of chronic colitis induced by dextran sulfate sodium (DSS) in mice but did not increase the ability of Treg cells to differentiate from the CD4^+^ T-cell population (82). Treatment with *A. muciniphila* alleviates liver tissue damage by upregulating serotonin (5-HT) expression and enhances cognitive function through the upregulation of brain-derived neurotrophic factor (BDNF) expression in the gut–liver–brain axis (83). Currently, *A. muciniphila* supplementation has been established as a novel therapeutic strategy across multiple disease models and is considered safe for oral administration in humans, thereby earning recognition as a new generation of probiotic bacteria.

Additionally, the EFSA has verified the safety of pasteurized *A. muciniphila* as a new food item. Numerous studies indicate that live *A. muciniphila* exhibits comparable therapeutic effects to those of pasteurized *A. muciniphila*, with some evidence suggesting that the latter may even demonstrate superior efficacy (84–87). For instance, both pasteurized *A. muciniphila* and live *A. muciniphila* restore damaged intestinal structure, improve liver function, and reduce the number of Treg cells in the spleen, but pasteurized *A. muciniphila* is more effective at improving glucose tolerance (87). Pasteurized *A. muciniphila* significantly reduced fat accumulation and dyslipidemia, thereby ameliorating obesity-related comorbidities (30). Additionally, another study demonstrated that pasteurized *A. muciniphila* enhances GLP-1 production to improve glucose homeostasis and alleviate symptoms associated with T2D in mice (24). Interestingly, pasteurized *A. muciniphila* improves colitis-related tumorigenesis by regulating CD8 cytotoxic T lymphocytes (CTLs) (88). A recent report showed that pasteurized *A. muciniphila* improved symptoms associated with irritable bowel syndrome in mice by reducing colon hypersensitivity reactions while enhancing intestinal barrier function (89). These lines of evidence suggest that pasteurized *A. muciniphila* also has a therapeutic effect on some diseases and is hopeful of clinical application.

#### 2.2.2 The outer membrane protein of *A. muciniphila*

Amuc_1100, a particular outer membrane protein extracted from *A. muciniphila*, was first isolated and characterized in 2017 (30). It has been demonstrated to interact with Toll-like receptor 2 (TLR2) and slightly promote glucagon-like peptide-1 (GLP-1) production via L cells, thereby enhancing intestinal microecology and improving metabolic outcomes in murine models of obesity and diabetes (30, 90). Furthermore, Amuc_1100 participates in the host intestinal immune response by activating TLR2 and TLR4 to produce IL-10 (91). Amuc_1100 demonstrates an antitumor effect in lung adenocarcinoma by inhibiting the JAK/STAT signaling pathway and enhancing both the quantity and activity of CD8^+^ T cells (79). Both *A. muciniphila* and Amuc_1100 ameliorate colitis by diminishing the infiltration of macrophages and CTLs in the colon; however, they also impede tumor progression by enhancing the population of CTLs within this tissue (88). Simultaneously, *A. muciniphila* and Amuc_1100 downregulate the expression of nodule receptor protein 3 (NLRP3) and TLR4/nuclear factor κB (NF-κB), inhibiting the production of inflammatory cytokines and thereby improving NAFLD (28). Furthermore, Amuc_1100 pretreatment attenuates the inflammatory response of acute pancreatitis by inhibiting the NF-κB signaling pathway, reducing the levels of proinflammatory cytokines, including TNF-α, IL-1β, IFN-γ, and IL-6, in both the spleen and pancreas, and decreasing the inflammatory infiltration of Ly6C^+^ macrophages and neutrophils within the spleen (92). Moreover, Amuc_1100 has been demonstrated to promote the proliferation of anti-inflammatory M2 macrophages while reducing the number of proinflammatory M1 macrophages in periodontitis, particularly by increasing IL-10 levels and decreasing TNF-α levels (93). Interestingly, a nanodrug formulation containing Amuc_1100 has been demonstrated to mitigate doxorubicin-induced cardiotoxicity by enhancing the intestinal barrier function, enhancing the synthesis of butyric and valeric acids, and ultimately regulating lymphocyte homeostasis in both the spleen and heart (94). Therefore, Amuc_1100 could become a new option for metabolic diseases, inflammation-related diseases, and immunotherapy. Furthermore, in 2022, research demonstrated that a phospholipid derived from the cell membranes of *A. muciniphila* exhibits immunomodulatory properties and facilitates the release of TNF-α and IL-6 (95). These findings are solely derived from *in-vitro* trials, and the outcomes of *in-vivo* research remain ambiguous; therefore, an in-depth investigation is required to verify these findings.

#### 2.2.3 The secreted protein of *A. muciniphila*

P9, an 84-kDa protein, was initially identified as being released through *A. muciniphila*, which significantly stimulates GLP-1 production and enhances thermogenesis in brown adipose tissue, thereby ameliorating glucose regulation in mice fed a high-fat diet (HFD) (96). Moreover, the underlying mechanism involves the binding of P9 to intercellular adhesion molecule 2 (ICAM-2), leading to the activation of the glucagon-like peptide-1 receptor (GLP-1R) pathway and IL-6. In a distinct study, recombinant *Lactococcus lactis* NZP9 was shown to secrete P9 heterologously, thereby stimulating NCI-H716 cells to produce GLP-1 as well (97). Furthermore, threonyl-tRNA synthetase (AmTARS) derived from *A. muciniphila* serves an essential function in monitoring and regulating immune response. AmTARS facilitates the polarization of M2 macrophages and engages in specific interactions with TLR2 (98). This interaction focuses on cAMP response element-binding (CREB), ultimately resulting in the production of IL-10. Concurrently, AmTARS promotes the restoration of IL-10-positive macrophages, elevates serum IL-10 levels, and mitigates inflammation in colitis mice (98). In a related study published this year, it was demonstrated that the protein Amuc_1409, secreted by *A. muciniphila*, serves an essential function in enhancing intestinal health through the modulation of intestinal stem cell (ISC) activity (52). Mechanistically, Amuc_1409 promotes the dissociation of the E-cadherin/β-catenin complex via its interaction with E-cadherin, thereby activating Wnt/β-catenin signaling and regulating ISC activity (52). Notably, Amuc_1409 significantly increases ISC populations across diverse experimental models, including isolated intestinal organoids and instances of chemically induced intestinal damage as well as natural aging in male mice.

#### 2.2.4 The extracellular vesicles of *A. muciniphila*

AmEVs, another component of *A. muciniphila*, demonstrate decreased levels in T2D patients and DSS mice models (99). Furthermore, in HFD-induced diabetic mice, AmEVs enhanced intestinal tight junction function and reduced the intestinal permeability of lipopolysaccharide (LPS)-treated Caco-2 cells (99). Similarly, AmEVs restored the gut microbiota of HFD/carbon tetrachloride (CCL4) mice to levels comparable to those of normal mice, improved intestinal permeability, reduced serum levels of TNF-α and IL-6, and elevated IL-10, thereby ameliorating liver damage and fibrosis in HFD/CCL4 mice (100). AmEVs can function as immunotherapeutics to impede the progression of prostate cancer, primarily by activating the effector state of CD8^+^ T cells and facilitating the polarization of macrophages toward the M1 phenotype (101). AmEVs have also demonstrated protective effects against hypertension; specifically, they enhance the expression of genes involved in vasoconstriction and vasodilation within the kidney, while simultaneously increasing the number of Treg cells and tending to reduce the production of proinflammatory cytokines (102, 103). On the other hand, AmEVs have also demonstrated potential for treating preeclampsia (PE) through AmEV transfer from the gastrointestinal tract to the placenta (104). Molecular investigations indicate that the therapeutic efficacy of AmEVs in managing PE is mediated through activation of the epidermal growth factor receptor (EGFR)-phosphoinositide 3-kinases (PI3K)/protein kinase B (AKT) signaling, thereby regulating trophoblast invasion into spiral arteries (SpA). Moreover, Amuc_2172, a recently identified probiotic enzyme derived from *A. muciniphila*, is present in AmEVs secreted by this bacterium. Research has demonstrated that Amuc_2172 functions as an acetyltransferase on Lys14 of histone H3 (H3K14ac), thereby enhancing the transcription and secretion of heat shock protein 70 (HSP70) and subsequently increasing both the quantity and activity of CTLs in colorectal cancer (CRC) (105). Finally, the researchers developed a bioengineering method to prepare macrophage membrane-encapsulated coated Amuc_2172 (CoAmuc_2172), which was proven to be a safe and reliable CRC treatment strategy, improving the clinical transformation value of the research. In another study, Amuc_2172 was shown to induce polarization in M1 macrophages, but puerarin reversed this change, thereby improving ulcerative colitis (106). Similarly, Amuc_1434, which is also derived from AmEVs, has been reported to enhance the adhesion of the colon cancer cell line LS174T that expresses high levels of mucin 2 (Muc2) and also facilitated the degradation of Muc2 in colon cancer cells (107). Subsequently, in-depth studies demonstrated that Amuc_1434 inhibits the viability of LS174T cells by the apoptotic pathway (108). The specific mechanism is that Amuc_1434 inhibited the proliferation and blocked the cell cycle of LS174T cells, promoted the apoptosis of CRC cell LS174T cells, and also induced the production of intracellular reactive oxygen species (ROS), damaged the mitochondrial membrane potential, and caused mitochondrial dysfunction of LS174T cells (108). Although AmEVs have demonstrated significant therapeutic potential for hypertension, gastrointestinal disorders, and tumor immunotherapy, its cultivation remains constrained as a derivative of *A. muciniphila*, with the inability to scale up production being a primary factor that impedes its development.

#### 2.2.5 The metabolites of *A. muciniphila*

Amuc_2109, a β-N-acetylhexosaminidase, is classified within the GH family 3 enzymes, secreted via *A. muciniphila*. Furthermore, Amuc_2109 exhibits enzymatic activity at pH levels above 4.0 and temperatures ranging from 20°C to 80°C, demonstrating its high survival rate in the intestinal environment (109). A recent study demonstrated that Amuc_2109 reduces the levels of TNF-α, IL-1β, and IL-6, as well as NLRP3 expression, while enhancing intestinal barrier function and improving DSS-induced colitis (110). SCFAs are the primary intestinal metabolites of *A. muciniphila*, with acetate, propionate, and butyrate as its principal components (111). SCFAs have been demonstrated to alleviate intestinal inflammation and cognitive dysfunction (112). *A. muciniphila* elevates serum levels of acetate and butyrate in sleep-deprived mice, while a mixture of SCFAs containing acetate and butyrate inhibits microglial synaptic phagocytosis and neuronal synapse loss, thereby ameliorating cognitive dysfunction induced by sleep deprivation (113). In addition, recent studies have shown that acetic acid derived from *A. muciniphila* plays an anti-aging role not only in *Caenorhabditis elegans* but also in naturally aging mice (114). On the other hand, studies have shown that acetic acid supports the growth and proliferation of tumor cells, leads to reduced infiltration of CD8^+^ T cells in tumor tissues, and promotes immune escape of tumor cells (115). Fucose fermentation enhances the production and metabolism of propionic acid in *A. muciniphila*, thereby facilitating the intestinal stem cell-mediated development of the intestinal epithelium (116). Other studies have shown that propionic acid plays an anti-inflammatory role and enhances intestinal barrier function by binding to the G-protein-coupled receptor 43 (GPR43) receptor (117, 118). Furthermore, the supplementation of butyric acid significantly mitigates pathological damage to lung tissue and improves the inflammatory response in mice with acute lung injury by restoring intestinal butyric acid production (119). Notably, *A. muciniphila* possesses the capability to synthesize γ-aminobutyric acid (GABA) in acidic environments, primarily through its genome encoding glutamate decarboxylase (120). In summary, the identification of these metabolites from *A. muciniphila* clarifies the relationship between *A. muciniphila* and health, elucidates the molecular mechanisms underlying its effects, and serves as a reference for future investigations into other probiotics.

### 2.3 Substances in traditional Chinese medicine that increase the abundance of *A. muciniphila*

Traditional Chinese medicine is abundant in natural bioactive compounds and demonstrates remarkable therapeutic efficacy by holistically regulating the homeostasis of various organs within the body (**Table 1**). Notably, numerous studies have indicated that traditional Chinese medicine can effectively restore intestinal barrier function and maintain microecological balance, mainly by growing the levels of *A. muciniphila* (121–123). Our prior research has shown that the number of *A. muciniphila* can be enhanced by 4.1-fold when cultured in a wolfberry decoction, and *in-vivo* experiments have further certified that wolfberry supplementation increases the abundance of *A. muciniphila* in both male and female mice (43). Further investigations demonstrated that a synergistic combination of wolfberry, yams, and chrysanthemums markedly increased the levels of *A. muciniphila* both *in vivo* and *in vitro*, with their polysaccharide mixtures displaying a similar effect (124). Furthermore, polysaccharides derived from *D. officinale*, *Atractylodes chinensis*, and *Poria cocos* promote the proliferation of intestinal microorganisms, including *Akkermansia*, *Lactobacillus*, and *Bifidobacterium* (45, 125, 126). In a mouse model of drug-induced liver injury, we similarly demonstrated that wolfberry significantly augmented the abundance of *A. muciniphila* by approximately twofold (127). Recent studies further demonstrated that wolfberry, *Platycodon grandiflorus* root extract, and *Polygonatum sibiricum* rhizome aqueous extracts enhance intestinal barrier function by elevating the abundance of *A. muciniphila* in HFD rats (128–130). *Hypericum perforatum* L. enhances the gut microbiome by elevating the levels of *A. muciniphila*, thereby contributing to an improvement in depressive symptoms (131). Interestingly, berberine is a promising prebiotic that indirectly elevates *A. muciniphila* levels by stimulating mucin secretion in the gut (44). Furthermore, traditional Chinese medicine prescriptions also address diseases associated with disruptions in intestinal homeostasis by enhancing the abundance of *A. muciniphila* (42, 132–135). Research has demonstrated that both Jinqi Jiangtang tablets and Shouhuitongbian can ameliorate T2D by enhancing the population of beneficial bacteria, particularly *A. muciniphila* (134, 135). Furthermore, the synergistic effect of *Astragalus membranaceus* and *Salvia miltiorrhiza* enhanced the number of *A. muciniphila* and improved diabetic nephropathy (122). Zexie-Baizhu decoction boosts the number of beneficial bacteria, especially *A. muciniphila*; regulates the composition of intestinal flora; and improves NAFLD (133). Shuangshen granules also increase the number of beneficial bacteria in the intestine, mainly *A. muciniphila* and *Limosilactobacillus reuteri*, while inhibiting the NF-κB pathway and thus inhibiting lung metastasis (132). The predominant dosage forms of traditional Chinese medicine are primarily oral. Upon entering the intestine, the active components of traditional Chinese medicine effectively interact with intestinal flora, which in turn influences the metabolic transformation of these compounds. This interaction may enhance the absorption of active ingredients, improve bioavailability, bolster intestinal barrier function, promote probiotic proliferation, and inhibit the growth of pathogenic bacteria. These mechanisms may constitute the primary pathways through which traditional Chinese medicine exerts its therapeutic effects. These results indicate the promise of traditional Chinese medicine as a prebiotic and provide new perspectives on its molecular mechanisms. In addition to the aforementioned factors, dietary interventions can also enhance the levels of *Akkermansia*, including dietary polyphenols and FODMAP (136). FODMAP refers to fermentable oligosaccharides, disaccharides, monosaccharides, and polyols, which are small carbohydrates that many people cannot digest. A randomized controlled trial demonstrated that oats possess prebiotic potential, significantly increasing the abundance of *A. muciniphila*, *Dialister*, *Butyrivibrio*, and *Paraprevotella* while concurrently reducing total cholesterol levels (137). Furthermore, a diverse array of compounds has been shown to significantly promote the growth of *A. muciniphila* in axenic culture, including metformin (138), tryptophan (139), fructo-oligosaccharides (22, 140), and betaine (141). Interestingly, the study demonstrated that the combination of probiotics *Lactobacillus rhamnosus* (*L. rhamnosus*) LMG S-28148 and *Bifidobacterium animalis* (*B. animalis*) LMG P-28149 significantly enhanced the abundance of *A. muciniphila*, likely attributable primarily to the influence of the latter (142). In contrast, the administration of *Bifidobacterium bifidum* G9-1 inhibited the growth of *A. muciniphila*, thus preventing the reduction of jejunal goblet cells in a mouse model of small intestinal loss (143). Noteworthy, broad-spectrum antibiotic treatment also increases the amount of *A. muciniphila* in the gut, especially vancomycin (144–146). While these findings collectively demonstrate an enhancement in the numbers of *A. muciniphila*, the underlying mechanisms of action differ among them, necessitating further investigation to determine any potential commonalities.

**Table 1:** Traditional Chinese medicine improves the abundance of *A. muciniphila*.

Drug	Research subject	Changes in flora	Ref.
Wolfberry	Male and female mice	*A. muciniphila* ↑	(43)
Wolfberry, yams, and chrysanthemums	Acetaminophen-induced liver injury in mice	*A. muciniphila* ↑	(124)
*Dendrobium officinale* polysaccharide	Cognitive dysfunction mice	*Akkermansia*, *Lactobacillus*, and *Bifidobacterium* ↑	(45)
*Atractylodes chinensis* polysaccharide	Alcohol-induced intestinal injury in rats	*A. muciniphila* ↑	(125)
*Poria cocos* polysaccharide	Male mice	*Akkermansia*, *Lactobacillus*, and *Bifidobacterium* ↑	(126)
*Platycodon grandiflorus* and *Polygonatum sibiricum*	High-fat diet-fed rats	*A. muciniphila* ↑	(129, 130)
*Hypericum perforatum* L.	Chronic restraint stress (CRS)-induced depression mice	*A. muciniphila* ↑	(131)
Berberine	High-fat diet-fed Institute of Cancer Research mice	*A. muciniphila* ↑	(44)
Jinqi Jiangtang tablet	Type 2 diabetes mellitus mice	*A. muciniphila* ↑, *Desulfovibrio* ↓	(134)
Shouhuitongbian	High-fat diet-fed mice and db/db mice	*Akkermansia* and *Parabacteroides* ↑	(135)
*Astragalus membranaceus* and *Salvia miltiorrhiza* in combination	Diabetic nephropathy rat model	*Akkermansia*, *A. muciniphila*, *Lactobacillus*, and *Lactobacillus murinus* ↑	(122)
Zexie-Baizhu decoction	High-fat diet-fed mice	*A. muciniphila* ↑	(133)
Shuangshen granules	Lewis lung cancer mouse model	*A. muciniphila* and *Limosilactobacillus reuteri ↑*	(132)

“↑” indicates that the abundance of relevant bacterial groups is increased. “↓” indicates that the abundance of relevant bacterial groups is reduced.

## 3 *A. muciniphila* and HCC

Intestinal flora constitutes a crucial component of the human intestinal microbial ecosystem and serves an essential function in liver physiology and pathology via the gut–liver axis (147). Numerous studies have shown that intestinal flora facilitates the onset and progression of HCC by influencing intestinal barrier function, exacerbating liver inflammation, and undermining antitumor responses. The primary factors implicated in this process include dysbiosis, increased intestinal permeability, microbe-associated molecular patterns (MAMPs), and their metabolites (147, 148). Therefore, dysregulation of the intestinal microbiota is a key factor in promoting the progression of HCC.

### 3.1 Changes in intestinal flora in HCC

**Figure 2:**
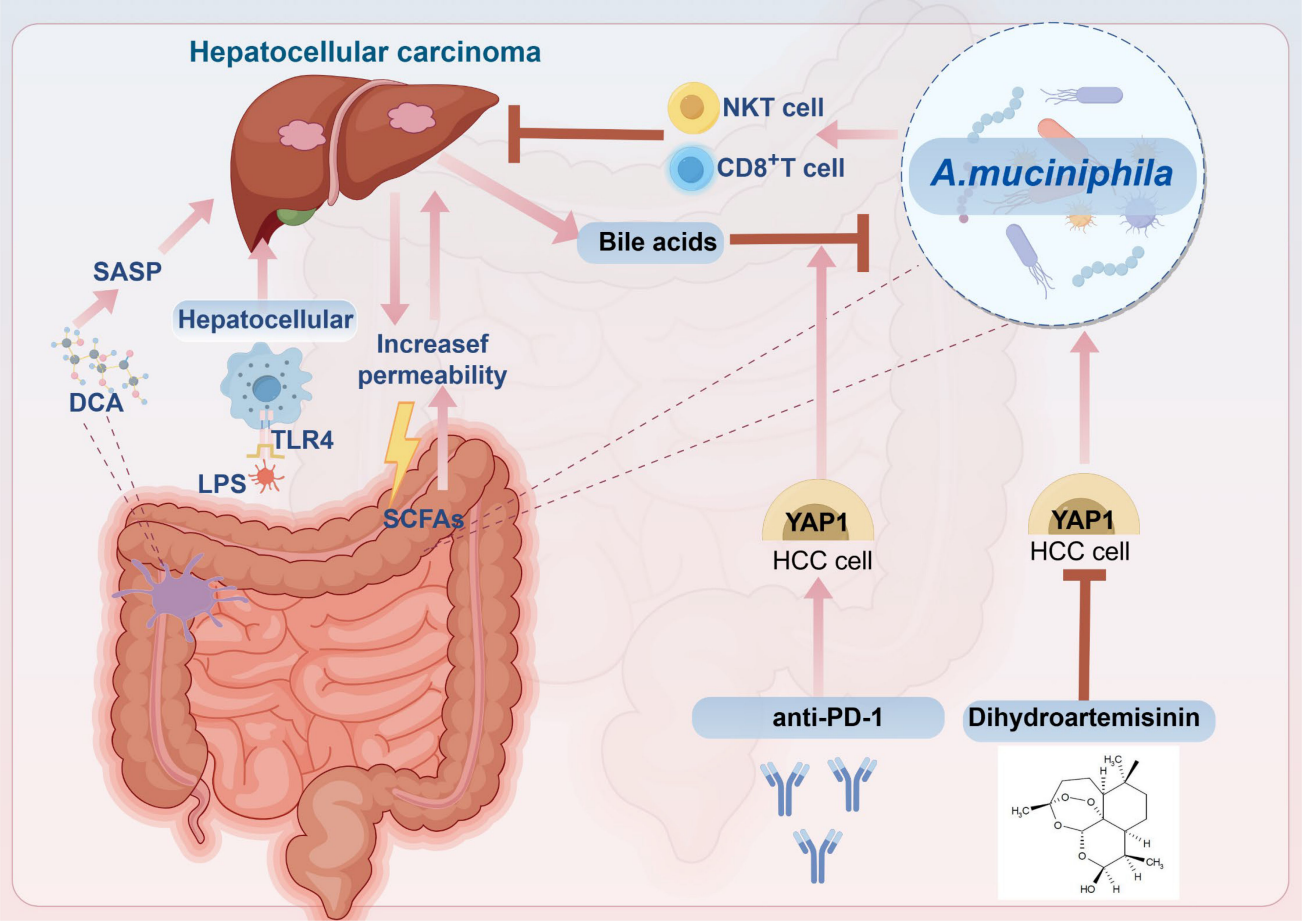
The correlation between hepatocellular carcinoma (HCC) and intestinal flora and the mechanism of *A. muciniphila* in treating HCC. Studies have shown that HCC promotes the increase of intestinal permeability, resulting in the production and release of large quantities of lipopolysaccharide (LPS). LPS binds to Toll-like receptor 4 (TLR4) on the surface of hepatocytes, leading to the production of various proinflammatory cytokines, thus promoting the occurrence and development of HCC. In addition, deoxycholic acid (DCA) derived from intestinal bacteria promotes the production of senescence-associated secretory phenotype (SASP), which leads to the progression of HCC. In our previous study, we found that anti-PD-1 promotes the expression of YAP1, thereby synergizing with bile acids to inhibit the growth of *A. muciniphila*, but dihydroartemisinin (DHA) can inhibit the expression of YAP1, thereby increasing the abundance of *A. muciniphila*, and the activity and number of CD8^+^ T cells sensitize the antitumor effect of anti-PD-1 in HCC.

Intestinal permeability is compromised at all stages of HCC development, allowing MAMPs to translocate from the intestine into the liver via the portal vein. This process promotes the onset and progression of HCC through signaling pathways mediated by receptors on hepatocyte surfaces (149). Furthermore, as HCC progresses, the biosynthetic pathways of LPS are upregulated, leading to a gradual increase in serum LPS levels during liver injury, which correlates with the Th1/Th17 proinflammatory phenotype (150). LPS, a common proinflammatory MAMP, constitutes the principal element of the outer membrane. LPS translocates into the bloodstream as a result of alterations in intestinal permeability, subsequently binding to TLR4 on hepatocyte surfaces. This interaction triggers the production of numerous inflammatory cytokines, thereby exacerbating the occurrence of liver cancer and facilitating hepatic cell regeneration (151–153). Studies found that high levels of LPS were detected in the peripheral blood of mice with N-nitrosodiethylamine (DEN)-induced HCC, and long-term application of low-toxic doses of LPS increased the volume and number of tumors in HCC (154). In another study, serum endotoxin levels were used as a relevant diagnostic biomarker for NAFLD and were used to detect and determine disease stage (155). The use of antibiotic regimens to reduce LPS or knock out the *TLR4* gene on hepatocytes in animal models of HCC to inhibit HCC growth confirmed the important role of the LPS–TLR4 axis in the occurrence and development of HCC (154). Metagenomic sequencing has elucidated alterations in the intestinal microbiota of patients with chronic liver disease and HCC, characterized by an addition in pathogenic bacteria and a reduction in beneficial bacterial populations (156, 157). For instance, among patients with liver cirrhosis, there was a notable rise in the abundance of *Enterobacter* within pathogenic taxa, while the numbers of *Lactobacillus* and *Akkermansia*, among beneficial taxa, were diminished (158, 159). Similarly, the abundance of *Phascolarctobcterium*, *Gemella*, *Enterococcus*, and *Streptococcus* increased in the intestinal flora of patients with HCC, and the levels of *Collinsella* and *Akkermansia* reduced (159). A separate study demonstrated that *A. muciniphila* was significantly diminished in both patients with non-alcoholic steatohepatitis (NASH)-associated HCC and in murine models (160). However, increased abundance of *A. muciniphila* has been reported in patients with esophageal adenocarcinoma and CRC (161–163). The level of *A. muciniphila* in tumor patients changes depending on the tumor site, but this does not affect the beneficial bacterial status of *A. muciniphila*. Supplementing *A. muciniphila* still inhibits tumor growth in HCC and CRC. In HCC induced by NASH, the proliferation of *Clostridium* in the intestine results in an increased synthesis of the bacterial metabolite deoxycholic acid (DCA) in the bloodstream. DCA primarily induces the production of the senescence-associated secretory phenotype (SASP) in hepatic stellate cells (HSCs) via enterohepatic circulation, which subsequently secretes various inflammatory and protumor factors within the liver or activates the mTOR signaling pathway, thereby facilitating the progression of NASH-induced HCC (149, 164). In addition to DCA, SCFAs not only serve as an energy source for intestinal epithelial cells but also modulate the permeability of the intestinal mucosa, resulting in increased harmful leakage within the intestine and thereby facilitating the progression of HCC (165, 166). Consequently, intestinal flora is anticipated to emerge as one of the most promising therapeutic targets for HCC. Finally, metagenomic analysis elucidated the correlation between species diversity and abundance within the intestinal microbiota and clinical responses as well as adverse reactions to immunotherapy. This study confirmed the potential of intestinal microbiota as a biomarker for liver cancer immunotherapy and identified new targets for modulating immunotherapy responses and mitigating adverse effects (167) (**Figure 2**).

### 3.2 *A. muciniphila* potentiates the efficacy of immunotherapy in HCC

Recent investigations indicate that the presence of specific intestinal microbiota and diverse bacterial populations is essential for effective antitumor immunotherapy (5, 167, 168). *A. muciniphila* has been demonstrated to enhance the response of tumor immunotherapy by inducing immunoglobulin G1 antibodies and promoting antigen-specific T-cell activation (169). Notably, research has shown that *A. muciniphila* is associated with favorable therapeutic outcomes in immunotherapy across various solid tumors, including HCC (11, 170), non-small cell lung cancer (NSCLC) (171), metastatic renal cell carcinoma (172), and CRC (173). Research has demonstrated that the response to ICIs influences the variety and structure of gut microbiota in fecal samples (11). Mice that responded improved the antitumor effectiveness of anti-PD-1 treatment, whereas FMT from non-responding mice did not yield comparable effects. Furthermore, oral administration of *A. muciniphila* improves the efficacy of anti-PD-1 in ICI non-responders, suggesting that *A. muciniphila* may play a potential role in regulating the clinical efficacy of ICIs (11). Therefore, increasing the abundance of *A. muciniphila* in the intestine or supplementing *A. muciniphila* appropriately is expected to become a new strategy for adjuvant HCC immunotherapy. Moreover, *A. muciniphila* effectively inhibited the progression of NASH to HCC, primarily by enhancing liver natural killer T (NKT) cell populations and reducing macrophage infiltration; additionally, *A. muciniphila* facilitated the cytotoxic activity of NKT cells against HepG2 cells (160). Furthermore, *A. muciniphila* enhances the efficacy of anti-PD-1 therapy in HCC mouse models by promoting apoptosis of HCC tumor cells and increasing the proportion of CD8^+^ T cells within the tumor microenvironment (TME) (170). In general, *A. muciniphila* exerts antitumor effects in HCC mainly by enhancing the number and activity of tumor killer cells and inhibiting the infiltration of immunosuppressive cells.

Similarly, supplementing *A. muciniphila* in the intestine enhanced the intestinal barrier function of mice, promoted intestinal homeostasis, improved antitumor immune function, and activated the activity of CD8^+^ T cells in the TME of ovarian cancer (174). Interestingly, fecal metagenomic analysis of patients with NSCLC or RCC who exhibited poor responses to anti-PD-1 antibody treatment revealed that those with partial response or stable disease had a higher abundance of *A. muciniphila* compared to patients experiencing disease progression (11). A recent study involving microbiome analysis of fecal samples from patients with advanced NSCLC demonstrated that *A. muciniphila* was associated with improved objective response rates and overall survival in multivariate analyses (175). Furthermore, utilizing 16S rDNA sequencing, it was observed that *A. muciniphila* facilitated an increase in its abundance within the bloodstream and tumor tissue, thereby inhibiting the progression of lung cancer in murine models (176). Another study demonstrated that the abundance of *A. muciniphila* is also reduced in patients with CRC. *A. muciniphila* induces the polarization of M1 macrophages via the TLR2/NLRP3 pathway, thereby inhibiting the progression of CRC (177). Likewise, *A. muciniphila* inhibits tryptophan metabolism through the aryl hydrocarbon receptor (AhR)/β-catenin signaling pathway to prevent the development of CRC (178). Moreover, the active components derived from *A. muciniphila* can also exhibit antitumor effects. AmEVs inhibit the growth of prostate cancer by enhancing the proportion of activated CD8^+^ T cells and M1 macrophages, while concurrently suppressing the polarization of M2 macrophages (101). Another *A. muciniphila*-derived component, Amuc_2172, inhibits the progression of CRC by enhancing the transcription and secretion of HSP70 to promote the immune response of CTLs (105). Both *A. muciniphila* and Amuc_1100 ameliorate colitis primarily by reducing the infiltration of macrophages and CTLs. Further investigations have demonstrated that they prevent colitis-related tumors by increasing the number of CTLs in the colon (88). Therefore, both *A. muciniphila* and its active compounds are candidate antitumor strategies, especially in tumor immunotherapy.

### 3.3 *A. muciniphila* potentiates the efficacy of chemotherapy drugs in cancer treatment

On the other hand, *A. muciniphila* can also improve the antitumor efficacy of chemotherapeutic drugs. For example, *A. muciniphila* enhances the antitumor effect of cisplatin in Lewis lung cancer mice, increasing the levels of IFN-γ, IL-6, and TNF-α by reducing the proportion of CD4^+^ CD25^+^ Foxp3^+^ Treg in the peripheral blood and spleen of mice (81). Studies have shown that after FOLFOX (oxaliplatin, fluorouracil, and calcium folinate) intervention, the abundance of *A. muciniphila* increases significantly and is positively correlated with treatment effect (179). It can also improve the antitumor effect of FOLFOX on colon cancer. Furthermore, pentadecanoic acid derived from *A. muciniphila* inhibits glycolysis by antagonizing the activity of far upstream element binding protein 1 (FUBP1), thereby enhancing the sensitivity of gastric cancer to oxaliplatin (180). These findings expand the potential applications of *A. muciniphila* and further substantiate its anticancer effects.

### 3.4 Traditional Chinese medicine combats tumors by enhancing the abundance of *A. muciniphila*

Likewise, interventions utilizing traditional Chinese medicine to enhance *A. muciniphila* colonization in the host represent a promising strategy for cancer treatment. Our previous study demonstrated that DHA inhibits YAP1 expression in a mouse model of liver cancer and enhances the accumulation of *A. muciniphila* to improve the therapeutic response to PD-1 inhibitor treatment (78). The combination of *A. muciniphila* with DHA, followed by its combination with the PD-1 inhibitor, resulted in an increase in the quantity and activation of CD8^+^ T cells within liver tumors. Quercetin combined with anti-PD-1 antibodies increases the levels in *Dubosiella* and *Akkermansia* and improves intestinal flora and macrophage immunity, thereby reshaping the TME of HCC (181). Furthermore, Shuangshen particles inhibit lung metastasis of lung cancer by increasing the abundance of *A. muciniphila*, enhancing the polarization of tumor-associated macrophages (TAMs), and suppressing the activation of the NF-κB signaling pathway (132). Similarly, another article showed that Huoxue Yiqi Recipe-2 increased *A. muciniphila* levels and thus enhanced the therapeutic effect of PD-L1 antibodies, thereby inhibiting the growth of lung cancer (182). Sini decoction inhibits the onset and progression of colitis-associated colon cancer in mice by upregulating the numbers of *A. muciniphila*, *Bifidobacterium* in the intestine, and CD8^+^ T cells, while downregulating CD4^+^ T cells, IL-6, and TNF-α (183). In addition, studies have shown that intervention with ginsenoside compound K restores intestinal flora to normal, partially increasing the abundance of *A. muciniphila* to prevent the progression of colitis-related colon cancer (184). It is reported that ginsenoside Rh4 also inhibits the progression of CRC by increasing the diversity of intestinal flora, especially the abundance of *A. muciniphila*, improving intestinal microbiota disorders and impaired intestinal barrier function caused by CRC (185). In short, traditional Chinese medicine and its active ingredients can treat cancer by increasing the colonization of *A. muciniphila*.

## 4 Conclusions

To sum up, as a standard for the next generation of probiotics, *A. muciniphila* is colonized in the intestinal mucus layer, participates in maintaining intestinal homeostasis and immune regulation to maintain host health, and is negatively related to the progress of various diseases, including metabolic-related diseases and tumors. On the contrary, studies have shown that *A. muciniphila* can also promote the progression of certain diseases. The mechanism behind this still needs further exploration and analysis to provide a reference for a detailed evaluation of *A. muciniphila* clinical application. In addition, the existing technology cannot cultivate *A. muciniphila* on a large scale. For *A. muciniphila* to be used on a large scale, the cultivation of *A. muciniphila* is a limitation. Although it has been shown that certain compounds, traditional Chinese medicines, and their monomers can enhance the levels of *A. muciniphila*, research on large-scale cultivation has not been reported, and the technology for large-scale preparation of *A. muciniphila* still needs further exploration. At present, most research on *A. muciniphila* focuses on *in-vitro* and rodent experiments, so clinical applications still require a lot of research, especially on side effects and safety. Although existing studies have displayed that oral *A. muciniphila* is safe and effective (30, 186, 187), it has not been officially approved as a food additive in China. In addition, the European Food Safety Authority has also verified the safety of pasteurized *A. muciniphila* as a new food. There are many studies showing that live *A. muciniphila* has the same therapeutic effect as pasteurized *A. muciniphila*, and even pasteurized *A. muciniphila* shows better effects (84–87), and other compounds derived from *A. muciniphila* have also shown therapeutic effects on certain diseases, indicating that research on the active ingredients of *A. muciniphila* is promising and further studies are needed. In terms of tumor immunotherapy, *A. muciniphila* has demonstrated strong immune activity and sensitizing effect, which provides favorable evidence for the use of *A. muciniphila* in tumor treatment. Overall, *A. muciniphila* is a powerful target for disease intervention, especially in tumor immunotherapy.
